# The Latest Developments in Spaceborne High-Resolution Wide-Swath SAR Systems and Imaging Methods

**DOI:** 10.3390/s24185978

**Published:** 2024-09-14

**Authors:** Ruizhen Song, Wei Wang, Weidong Yu

**Affiliations:** 1 Department of Space Microwave Remote Sensing System, Aerospace Information Research Institute, Chinese Academy of Sciences, Beijing 100090, China; songruizhen20@mails.ucas.ac.cn (R.S.); wwang@mail.ie.ac.cn (W.W.); 2 School of Electronic, Electrical and Communication Engineering, University of Chinese Academy of Sciences, Beijing 100049, China

**Keywords:** azimuth multichannel technique, digital beamforming technique, high-resolution wide-swath, pulse repetition interval variation technique, synthetic aperture radar

## Abstract

Azimuth resolution and swath width are two crucial parameters in spaceborne synthetic aperture radar (SAR) systems. However, it is difficult for conventional spaceborne SAR to simultaneously achieve high-resolution wide-swath (HRWS) due to the minimum antenna area constraint. To mitigate this limitation, some representative HRWS SAR imaging techniques have been investigated, e.g., the azimuth multichannel technique, digital beamforming (DBF) technique, and pulse repetition interval (PRI) variation technique. This paper focus on a comprehensive review of the three techniques with respect to their latest developments. First, some key parameters of HRWS SAR are presented and analyzed to help the reader establish the general concept of SAR. Second, three techniques are introduced in detail, roughly following a simple-to-complex approach, i.e., start with the basic concept, then move to the core principles and classic technical details, and finally report the technical challenges and corresponding solutions. Third, some in-depth insights on the comparison among the three techniques are given. The purpose of this paper is to provide a review and brief perspective on the development of HRWS SAR.

## 1. Introduction

Synthetic aperture radar (SAR) is an important spaceborne microwave remote sensing technology with global, all-day, all-weather Earth observation capability [1]. Geometric resolution and swath width are two crucial parameters in spaceborne SAR systems. On the one hand, high-resolution SAR images can provide more detailed information of the target, which is conducive to target identification and feature extraction. On the other hand, wide swath enables SAR to obtain broader scene information, which is beneficial in reducing the revisit cycle for specific areas. Driven by the spaceborne missions of scientific and commercial applications, e.g., high-precision interferometry, polarization interferometric inversion, and hazard and disaster monitoring, higher resolution and wider swath are both urgently required. However, due to the minimum antenna area constraint, there is an inherent trade-off between high resolution and wide swath in conventional spaceborne SAR, i.e., a challenge is presented in obtaining high-resolution wide-swath (HRWS) SAR images [2]. To break through the above dilemma, a series of advanced spaceborne HRWS SAR systems and imaging methods have been successively proposed in past decades. Building on the development of existing HRWS SAR and following the core ideas behind it, this paper reviews some representative technologies as follows.

In 1992, the concept of a single-phase-center multiple-beam (SPC-MB) system was proposed by Currie et al. [3], which can be further divided into a single-phase-center multiple-elevation-beam (SPC-MEB) system and single-phase-center multiple-azimuth-beam (SPC-MAB) system. The former uses a wide beam in elevation to illuminate a wide coverage and adopts a high pulse repetition frequency (PRF) for a fine azimuth resolution. Then, multiple sub-beams in elevation are formed and the echoes from several sub-swaths are separated. But this operating mode leads to serious range ambiguity due to the small intervals between sub-swaths. The latter adopts a wide beam in azimuth for a high azimuth resolution and a low PRF is chosen for wide swath. Then, multiple narrow beams in azimuth with different pointing directions are formed for reception. Further, the sub-echo signals are spectrally spliced to obtain a large Doppler bandwidth for a high azimuth resolution. However, SPC-MAB also suffers from severe azimuth ambiguity due to the interference between the sub-beams. To suppress the azimuth ambiguities, the concept of displaced phase center multiple azimuth beam (DPC-MAB) is proposed [3]. It also transmits a wide beam in azimuth for a high azimuth resolution, but differs from SPC-MAB in that it uses multiple channels linearly spaced in an along-track (azimuth) manner for reception. Such an operating mode can equivalently increase the azimuth samples, thus obtaining a significantly increased swath width by reducing the system’s actual PRF without sacrificing the azimuth resolution. Due to its advantages, the DPC-MAB technique, i.e., the azimuth multichannel technique, has been successfully applied to in-orbit spaceborne SAR systems such as TerraSAR-X [4], RadarSat-2 [5], GaoFen-3 [6], and LT-1 [7,8].

After that, Callaghan et al. proposed a four-array SAR mode based on DPC-MAB, where two phase centers in both azimuth and elevation are employed [9]. The four-array SAR system adopts a small-aperture antenna to generate a two-dimensional wide transmit beam and four sub-apertures to receive the echo simultaneously. The joint processing of the two receiving channels in azimuth can suppress the azimuth ambiguity, reduce the requirement of the PRF and then increase the swath width; the two receiving channels in elevation use the digital beamforming (DBF) technique, which generates a null-steering in the direction of the ambiguity echoes to suppress the range ambiguity. The four-array system can improve the swath width by nearly four times without the loss of azimuth resolution. Moreover, Suess et al. presented an HRWS SAR imaging mode by following the similar core ideal of the four-array system [10]. This mode still employs a small-aperture antenna to achieve wide coverage and utilizes a two-dimensional multichannel to receive the echo. The azimuth spectrum aliasing caused by undersampling of the actual PRF can be eliminated by azimuth multichannel reconstruction. The scan-on-receive (SCORE) technique in elevation is utilized to form a high-gain pencil beam following the echo in real time, which can compensate for the gain loss due to the small transmit aperture area and effectively suppress the range ambiguity. Furthermore, Krieger et al. proposed a multidimensional waveform coding technique to realize HRWS SAR imaging [11]. In this mode, the transmit pulse is divided into multiple sub-pulses. For azimuth multidimensional waveform coding, multiple beams corresponding to multiple sub-pulses sequentially illuminate the same swath, and multiple DBF networks are used to simultaneously generate high-gain narrow beams in elevation, which point in different directions to separate the echoes of different sub-pulses. Then, a high azimuth resolution can be realized by splicing the azimuth spectrum of the sub-echo signal. Also, the azimuth ambiguity can be suppressed by azimuth multichannel reconstruction, which reduces the requirement of the system PRF and helps to improve the swath width. For elevation multidimensional waveform coding, multiple sub-pulses illuminate the interested swath from a far to near range to compress the echo window length. Similarly, multiple DBF networks are used for echo separation and range ambiguity suppression. Then, the sub-swaths are spliced together to obtain a wide-swath SAR image. It is not difficult to find that a core idea of increasing the spatial freedom of the SAR system in elevation emerges in the above advanced imaging modes. This core idea is implemented by combining the real-time digital beamforming technique with multiple independent receive channels equipped in elevation, which is also known as the elevation DBF technique. Although DBF as a functional technique does not directly mitigate the minimum antenna area constraint, there is no doubt that it can significantly improve the sensitivity and range ambiguity performance of the SAR system. In addition, it also provides the SAR system with the opportunity to distinguish the elevation angles of the echoes. Thanks to these advantages, the DBF technique in elevation has become a key technology to support future spaceborne SAR to realize HRWS imaging.

Although wide-swath SAR images can be obtained by multibeam and DBF techniques, blind ranges with fixed positions appear in the swath due to the inability to receive echoes when transmitting pulses, thus resulting in discontinuous imaging. To overcome this problem, a multiple-elevation-beam (MEB) ScanSAR mode is proposed. In this mode, multiple sub-swaths are illuminated simultaneously at a Burst time, and different pulse repetition intervals (PRIs) are selected at different Burst times to stagger the location of the blind ranges [12,13]. Following this idea of periodically varying the PRI to stagger the blind ranges, a strip mode with continuous PRI variation is proposed, where a wide beam is used to illuminate the entire swath and the continuously varied PRI is used to shift the blind ranges [14,15]. Subsequently, Villano et al. named this mode based on the PRI variation technique the staggered imaging mode, which in combination with the DBF technique in elevation can realize ultrawide continuous swath imaging without loss of azimuth resolution [16]. The PRI variation technique breaks the limitation of the pulse system and allows reception across multiple PRIs, which is a key technique for realizing high-resolution ultrawide continuous swath SAR imaging in the future.

Considering the important role and significant potential of the azimuth multichannel technique, DBF technique, and advanced PRI variation technique in the development of HRWS SAR, and the fact that they have already been validated by engineering or are about to be validated [17,18,19], this paper focuses on a detailed review of these three techniques.

## 2. Analysis of Constraints for HRWS Spaceborne SAR

Spaceborne SAR is not an ideal sensor and its imaging performance is limited by system characteristics and inherent constraints. In this section, several constraints that limit the ability of the spaceborne SAR system to achieve HRWS imaging are analyzed, including the minimum antenna area constraint, the echo timing constraint, and some critical system performance constraints.

### 2.1. Minimum Antenna Area Constraint

Azimuth resolution and swath width are two of the most important performance parameters and represent the imaging capability of a SAR system. Azimuth resolution represents the ability of SAR to distinguish between two adjacent targets on the ground that are distributed along the azimuth. According to the theory of SAR imaging, azimuth resolution is related to the SAR platform speed and the Doppler bandwidth in azimuth [1]. Then, based on the relationship between the Doppler bandwidth and beamwidth, the azimuth resolution can be further expressed as

(1) $$\rho_{a}=\frac{\psi_{a}\cdot v_{s}}{B_{d}}=\frac{\psi_{a}\cdot L_{a}}{2}$$

where $\psi_{a}$ is the spreading factor, $v_{s}$ is the platform speed, and $B_{d}$ and $L_{a}$ are the Doppler bandwidth and antenna length, respectively. The swath width is defined as the width of the ground range corresponding to the area illuminated by the beam during SAR operation. Assuming that the incidence angle at the center of the interested swath is $\alpha_{c}$, corresponding to a slant range of $R_{c}$, the transmission wavelength is λ and the height of the antenna and the beamwidth in elevation are $L_{r}$ and $\theta_{bw}$, respectively. Then, the swath widths in ground range and slant range can be expressed as (2) and (3), respectively.

(2) $$W_{g}=\frac{\theta_{bw}\cdot R_{c}}{\cos\left(\alpha_{c}\right)}=\frac{\lambda \cdot R_{c}}{L_{r}\cdot \cos\left(\alpha_{c}\right)}$$

(3) $$W_{r}=W_{g}\cdot \sin\left(\alpha_{c}\right)$$

The SAR operates in pulsed mode, where the echo of the same transmitted pulse is required to be received in a PRI. In this case, the PRI is required to satisfy

(4) $$\mathrm{PRI}>\frac{2\left(R_{\max}-R_{\min}\right)}{c}=\frac{2W_{r}}{c}$$

where $R_{\min}$ and $R_{\max}$ are the nearest and farthest slant range of the interested swath, respectively. *c* is the speed of light and PRI=1/PRF. Then, (4) can be derived as

(5) $$W_{r}<\frac{c}{2\mathrm{PRF}}$$

Since the echo signal is sampled by the PRF in azimuth, to ensure that the azimuth spectrum of the sampled signal is not severely aliased, it is required to satisfy

(6) $$\mathrm{PRF}>B_{d}=\frac{v_{s}}{\rho_{a}}$$

According to (5) and (6), the PRF is required to be as small as possible to achieve a wide swath, but as large as possible to achieve a high azimuth resolution. The above different requirements on the PRF lead to a contradiction between the azimuth resolution and swath width of a conventional single-channel SAR. Further, the relationship between swath width and azimuth resolution can be obtained as follows

(7) $$\frac{W_{r}}{\rho_{a}}<\frac{c}{2v_{s}}$$

Substituting (1)–(3) into (7), the minimum antenna area constraint can be derived as

(8) $$A_{\mathrm{ant}}=L_{a}\cdot L_{r}>\frac{4\lambda v_{s}R_{c}\tan\left(\alpha_{c}\right)}{c}$$

where $A_{\mathrm{ant}}$ represents the antenna area. As can be seen from the above formula, when the wavelength, platform velocity, slant range, and incidence angle of the SAR are given, the lower limit of the antenna area is determined.

### 2.2. Echo Timing Constraint

**Figure 1:**
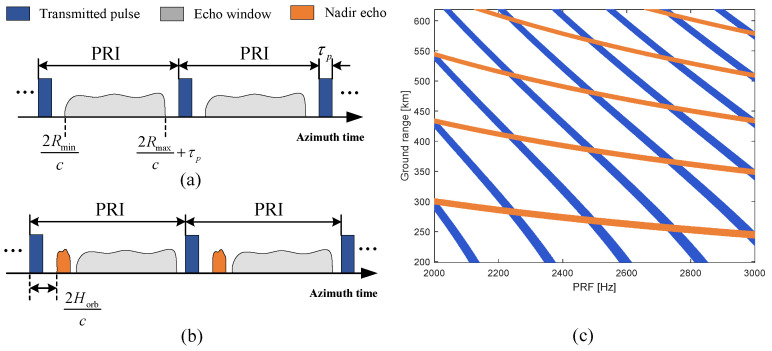
Schematic of echo timing constraint for spaceborne synthetic aperture radar (SAR). (**a**) Timing diagram of pulse transmitting and receiving to avoid transmission blockage, where the horizontal axis indicates azimuth time. (**b**) Timing diagram of pulse transmitting and receiving to avoid nadir echo interference. (**c**) Timing (diamond) diagram due to transmission blockage (blue strips) and nadir echo (orange strips) versus pulse repetition frequency (PRF).

For a spaceborne SAR system using a transceiver antenna, there are typically two constraints on the echo timing. On the one hand, since the transmit and receive pulses are separated by multiple PRIs, the echo window usually needs to avoid interference from the transmitted pulse, as shown in Figure 1a. On the other hand, the nadir echo power is usually high, which can result in severe range ambiguity; thus, the nadir echo also needs to be avoided, as shown in Figure 1b. According to the timing diagram of pulse transmission and reception for the spaceborne SAR shown in Figure 1, to avoid transmission blockage and nadir echo interference, the constraints on the echo window timing can be expressed as follows:

(9) $$\left\{\begin{array}{c} \mathrm{Int}\left(2R_{\min}\cdot \mathrm{PRF}/c\right)=\mathrm{Int}\left(2R_{\max}\cdot \mathrm{PRF}/c\right)\\ \mathrm{Frac}\left(2R_{\min}\cdot \mathrm{PRF}/c\right)/\mathrm{PRF}>\tau_{p}\\ \mathrm{Frac}\left(2R_{\max}\cdot \mathrm{PRF}/c\right)/\mathrm{PRF}<1/\mathrm{PRF}-\tau_{p} \end{array}\right.$$

(10) Int2Rmin−Horb·PRF/c=Int2Rmax−Horb·PRF/cFrac2Rmin−Horb·PRF2Rmin−Horb·PRFcc/PRF>τpFrac2Rmax−Horb·PRF2Rmax−Horb·PRFcc/PRF<1/PRF−τp

where (9) and (10) are the constraints caused by transmission blockage and nadir echo interference, respectively. $\mathrm{Int}\left(\cdot \right)$ and $\mathrm{Frac}\left(\cdot \right)$ are the integer and fraction functions, respectively. $\tau_{p}$ is the pulse duration and $H_{\mathrm{orb}}$ represents the orbit height.

Then, according to (9) and (10), an example of the echo window timing constraint is shown in Figure 1c, where the orbit height is 600 km and pulse duration is 30 μs. The blue and orange strips indicate the transmission blockage and nadir echo locations, respectively, and the white areas are effective illumination areas and PRF values. It can be seen that as the azimuth resolution improves, the PRF needs to be increased, but this results in the selectable swath becoming narrower. Therefore, it is difficult to improve both azimuth resolution and swath width simultaneously.

### 2.3. Critical Performance of Spaceborne SAR System

To satisfy the requirements of SAR users, spaceborne SAR is also constrained by system performance. This subsection mainly introduces the critical performance of the spaceborne SAR system, including the azimuth ambiguity-to-signal ratio (AASR), range ambiguity-to-signal ratio (RASR), and noise equivalent sigma zero (NESZ).

#### 2.3.1. AASR

**Figure 2:**
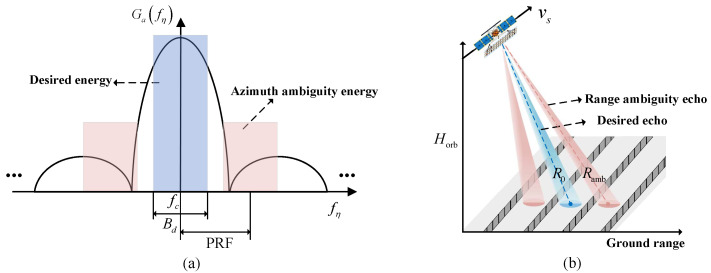
Schematic of ambiguity for spaceborne SAR. (**a**) Schematic of azimuth ambiguity, where the horizontal axis represents the Doppler frequency and the vertical axis represents the azimuth antenna pattern. (**b**) Schematic of range ambiguity, where the horizontal axis represents the ground range, the vertical axis represents the orbit height, the upper axis of the figure shows the direction of platform movement, and the black stripes are the blind ranges caused by the transmission blockages.

The azimuth ambiguity in SAR arises from the azimuth antenna pattern. The echo of SAR is sampled at the PRF in azimuth; thus, the azimuth signal is discrete. This requires the azimuth signal to be band-limited, with a bandwidth smaller than the PRF. However, the Doppler bandwidth of the azimuth signal is not strictly limited to $B_{d}$ in practice. Therefore, when sampling at the PRF, signals with Doppler frequencies that are integer multiples of the PRF in the echo will be extended into the main lobe, resulting in aliasing of the main lobe signal, which causes azimuth ambiguity in SAR. Figure 2a gives a schematic of azimuth ambiguity, where $f_{c}$ is the centre frequency of the signal, $f_{\eta }$ is the Doppler frequency, and $G_{a}$ is the azimuth antenna pattern.

Based on the above principle, the equation for estimating the AASR of a SAR system can be expressed as

(11) $$AASR=\frac{\sum_{p=-\infty ,p\neq 0}^{p=+\infty }\int_{f_{c}-B_{d}/2}^{f_{c}+B_{d}/2}{G_{a}}^{2}\left(f_{\eta }+p\cdot PRF\right)df_{\eta }}{\int_{f_{c}-B_{d}/2}^{f_{c}+B_{d}/2}{G_{a}}^{2}\left(f_{\eta }\right)df_{\eta }}$$

where *p* is the order of azimuth ambiguity. It can be seen that the AASR performance of SAR will benefit from a high PRF.

#### 2.3.2. RASR

The antenna pattern in the elevation of SAR is also not ideal, resulting in echoes outside the interested swath being mixed with desired echoes from the interested swath. This leads to range ambiguity and degradation of the SAR image quality.

The formation of range ambiguity is schematically illustrated in Figure 2b; the corresponding RASR evaluation formula is

(12) $$RASR=\frac{\sum_{q=-\infty ,q\neq 0}^{q=+\infty }\frac{\sigma \left(\alpha_{amb,q}\right){G_{e}}^{2}\left(\theta_{amb,q}\right)}{R_{amb,q}\cdot \sin\left(\alpha_{amb,q}\right)}}{\frac{\sigma \left(\alpha_{0}\right){G_{e}}^{2}\left(\theta_{0}\right)}{R_{0}\cdot \sin\left(\alpha_{0}\right)}}$$

where *q* is the order of range ambiguity and the corner markers 0 and amb denote the parameters corresponding to the desired and range ambiguity signals, respectively. α and θ are the incidence angle and look angle, respectively. *R* is the slant range and $G_{e}$ is the antenna pattern in elevation. In particular, $R_{amb,q}=R_{0}+q/PRF$; it can be seen that the RASR performance of SAR will benefit from a low PRF, which conflicts with the AASR.

#### 2.3.3. NESZ

The NESZ of SAR, also known as system sensitivity, is used to measure the ability of SAR to image weak targets. It represents the backscatter coefficient of the target when the output signal-to-noise ratio (SNR) of the SAR image is 0 dB. The evaluation expression for NESZ is as follows

(13) $$NESZ=\frac{256\pi^{3}R_{0}\cdot v_{s}\cdot \sin\left(\alpha_{0}\right)\cdot k_{b}\cdot T_{n}\cdot B_{r}\cdot L_{f}}{P_{t}\cdot PRF\cdot \tau_{p}\cdot {G_{e}}^{2}\left(\theta_{0}\right)\cdot \lambda^{3}\cdot c}$$

where $k_{b}$ is Boltzmann’s constant, $T_{n}$ is the system noise temperature, $B_{r}$ is the signal bandwidth, $L_{f}$ is the system loss, and $P_{t}$ is the peak power. It can be found that increasing the system PRF and/or pulse duration can improve the NESZ, but leads to a smaller echo window, which in turn reduces the swath width and deteriorates the RASR performance. Therefore, there is also a trade-off between improving the NESZ and other SAR system performance parameters.

## 3. Azimuth Multichannel Technique

This section provides a review of the azimuth multichannel technique. Section 3.1 gives the core principle of the azimuth multichannel technique. Section 3.2 introduces one of the major challenges faced by azimuth multichannel, i.e., azimuth multichannel signal reconstruction, and reviews a number of classic and novel solutions. Section 3.3 describes the problem of multichannel error calibration that needs to be addressed in the azimuth multichannel SAR system, and reviews a number of methods.

### 3.1. Principle of Azimuth Multichannel Technique

In recent years, azimuth multichannel has become an important technique for spaceborne SAR systems to realize HRWS imaging, where the antenna is divided into multiple channels along the azimuth. Generally, a wide beam is transmitted by an azimuth channel, and then the ground echoes are received simultaneously by a number of receiving channels along the azimuth, each of which is pointed to the same area. Echoes received from multiple channels are downconverted, digitally sampled, and then stored separately. with this approach, the SAR system can obtain multiple echo signals from different azimuth positions in a single PRI. Thus, the azimuth multichannel technique essentially compensates for the time-domain sampling by increasing the spatial-domain sampling. As a result, the PRF can be reduced without sacrificing the azimuth resolution, and correspondingly the swath width of spaceborne SAR can be significantly improved [3,20].

In an azimuth multichannel system, the transmit phase center and multiple receive phase centers are separated from each other; thus, the phase modulation of the multichannel echoes is different. The azimuth echo signal of the *i*th channel for the point target located at the slant range $R_{0}$ can be expressed as

(14) $$s_{i}\left(\eta \right)=\exp\left\{-j\frac{2\pi }{\lambda }\left(\sqrt{{R_{0}}^{2}+{\left(v_{s}\eta \right)}^{2}}+\sqrt{{R_{0}}^{2}+{\left(v_{s}\eta -d_{i}\right)}^{2}}\right)\right\}$$

where η is the azimuth time. $d_{i}$ is the distance between the phase center of the *i*th receive channel and that of the transmit channel. Taylor expansion of the phase in (14) to a quadratic term yields the following approximate expression

(15) $$s_{i}\left(\eta \right)=\exp\left\{-j\frac{4\pi }{\lambda }R_{0}\right\}\exp\left\{-j\frac{\pi d_{i}^{2}}{2\lambda R_{0}}\right\}\exp\left\{-j\frac{2\pi v_{s}^{2}}{\lambda R_{0}}{\left(\eta -\frac{d_{i}}{2v_{s}}\right)}^{2}\right\}$$

The azimuth signal of a single-channel SAR can be approximated as

(16) $$s\left(\eta \right)=\exp\left\{-j\frac{4\pi }{\lambda }R_{0}\right\}\exp\left\{-j\frac{2\pi v_{s}^{2}\eta^{2}}{\lambda R_{0}}\right\}$$

**Figure 3:**
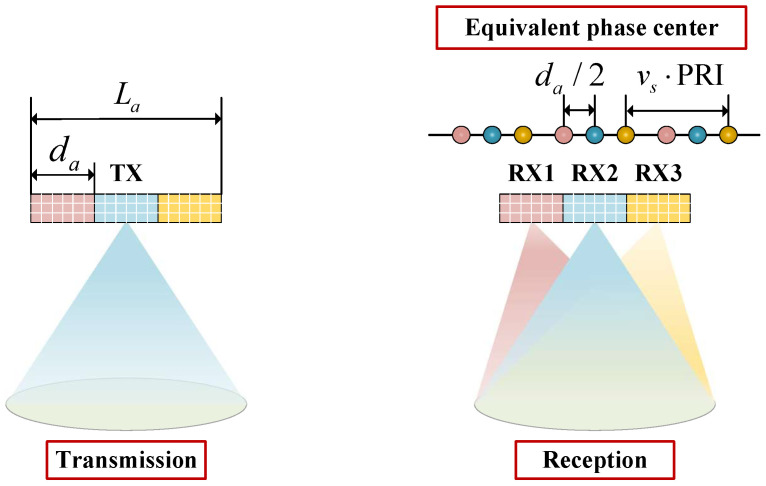
Schematic diagram of the operation principle for azimuth three-channel SAR, where different colors represent the echo signals of different channels.

Comparison reveals that after compensating for the constant phase of the second term in (15), the echo in the *i*th channel is approximately equivalent to the return in (16) at $d_{i}/2$. Taking the azimuth three-channel SAR as an example, its operation principle is shown schematically in Figure 3, where $d_{a}$ is the distance between neighboring channels. Obviously, for a SAR system with $N_{a}$ azimuth channels, $N_{a}$ equivalent azimuth samples can be obtained simultaneously within a single PRI.

In addition, as can be seen from Figure 3, when the azimuth multichannel system operates at an ideal PRF, i.e., $\mathrm{PRF}=2v_{s}/L_{a}$, uniform raw azimuth samples can be obtained. In this case, it is only required to perform constant phase compensation for each channel echo signal, and then arrange the multichannel samples according to the correlation between the channel phases to obtain SAR data equivalent to the conventional single-channel SAR. However, it is difficult for a real spaceborne SAR system to operate at a rigid selected PRF due to limitations such as nadir echoes and transmission blockages. Correspondingly, the multichannel raw samples are not uniformly distributed in azimuth, which leads to virtual targets in the imaging results when processed directly using conventional imaging algorithms. Therefore, it is necessary to investigate azimuth nonuniform signal reconstruction algorithms with wide applicability when the PRF deviates from the ideal value.

### 3.2. Azimuth Multichannel Processing Scheme

As mentioned above, the azimuth samples in azimuth multichannel SAR are generally nonuniform. Up to date, a large number of scholars have conducted extensive and in-depth research on multichannel signal reconstruction methods for azimuth multichannel SAR, which are reviewed in this subsection.

**Figure 4:**
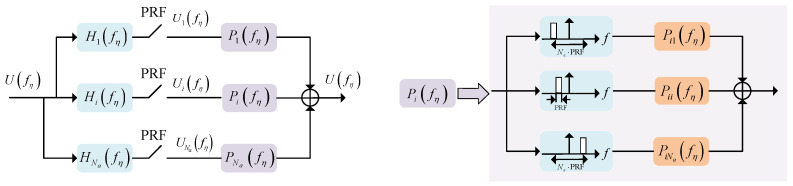
Principle schematic of azimuth multichannel signal reconstruction algorithm by reconstruction filters.

According to the generalized sampling theorem [21,22], when a signal with bandwidth $B_{d}$ is sampled using $N_{a}$ filters and each with a sampling rate not lower than $B_{d}/N_{a}$, the Doppler spectrum of the original signal can be recovered without ambiguity as long as the two samples do not overlap. Based on such a theory, Krieger et al. proposed the classical azimuth multichannel signal reconstruction algorithm without ambiguity for nonuniform sampling [20]. In this method, the reconstruction process of nonuniform sampling is regarded as the inverse process of azimuth multichannel data formation. The reconstruction filters can be derived from the transfer function of the pre-filters before data sampling. A principle schematic of this algorithm is given in Figure 4, where $U\left(f_{\eta }\right)$ represents the equivalent signal spectrum of single-channel SAR, $f_{\eta }$ is the Doppler frequency, and $U_{i}\left(f_{\eta }\right)$ is the signal spectrum of the *i*th channel after pre-filtering and undersampling. The reconstruction process of the azimuth multichannel signal is to rearrange the $N_{a}$ signals after passing through $N_{a}$ reconstruction filters $P_{i}\left(f_{\eta }\right)$ to obtain the oversampled single-channel signal. Comparing (15) and (16) yields the pre-filter for each channel, whose frequency domain form can be expressed as

(17) $$H_{i}\left(f_{\eta }\right)=\exp\left[-j\frac{\pi d_{i}^{2}}{2\lambda R_{0}}\right]\cdot \exp\left[-j2\pi \frac{d_{i}}{2v_{s}}f_{\eta }\right]$$

Correspondingly, the reconstruction filter bank can be expressed as

(18) $$P\left(f_{\eta }\right)=H^{-1}\left(f_{\eta }\right)=\left[\begin{array}{cccc} P_{11}\left(f_{\eta }\right) & P_{12}\left(f_{\eta }+\mathrm{PRF}\right) & \cdots & P_{1N_{a}}\left(f_{\eta }+\left(N_{a}-1\right)\mathrm{PRF}\right)\\ P_{21}\left(f_{\eta }\right) & P_{22}\left(f_{\eta }+\mathrm{PRF}\right) & \cdots & P_{2N_{a}}\left(f_{\eta }+\left(N_{a}-1\right)\mathrm{PRF}\right)\\ \vdots & \vdots & \ddots & \vdots \\ P_{N_{a}1}\left(f_{\eta }\right) & P_{N_{a}2}\left(f_{\eta }+\mathrm{PRF}\right) & \cdots & P_{N_{a}N_{a}}\left(f_{\eta }+\left(N_{a}-1\right)\mathrm{PRF}\right) \end{array}\right]$$

Based on this, Gebert et al. thoroughly investigated the effect of spectral reconstruction processing on the system SNR and azimuth ambiguity, then proposed a phase center adaptive method to optimize the SAR system performance [23]. Moreover, based on the idea of space-time adaptive processing (STAP) [24], Li et al. proposed an approach to reconstruct azimuth multichannel data by constructing an optimal beamformer [25]. The essentiality of this approach is that the overlapped Doppler spectrums are spatially distinguishable. To extract a Doppler unit in aliased spectrums, the main beam of the array response for the spatial domain filter is pointed at its corresponding frequency component, while null-steering beams are formed on the other spectrum components. The core of the spatial domain filtering approach is the construction of an optimal spatial domain filter, where the criterion is to minimize all the interference energy. Further, Sun et al. studied an improved post-Doppler STAP method, which can be applied to beam steering SAR [26]. Compared with the method of constructing a filter bank by the transfer function, the STAP uses the echo data to construct a space-time filter, which forms a null-steering beam by minimizing the noise and ambiguity energy through the optimization criterion. Thus, the latter can adaptively suppress system noise and multichannel errors, and is more adapted to the system with non-ideal channel errors. However, when the SNR of the scene is relatively low, the use of the spatial domain filtering weakens the suppression of ambiguous frequency components due to the suppression of noise. In this case, the filter bank reconstruction method is more able to obtain a better reconstruction performance.

It is not difficult to find that the STAP approach is similar in principle to the classical Capon beamformer, in which the output signal power is required to be minimized when the beam is generated [27]. Inspired by this, a new criterion can be set up to make the SAR system perform better in other aspects. For example, Zhang et al. introduced the robust Capon beamformer to effectively improve the tolerance and robustness of various types of errors during the azimuth multichannel data reconstruction process [28]. Some other solutions to signal reconstruction based on the optimization of specific cost functions have also been reported, i.e., the maximum signal-to-ambiguity-plus-noise ratio method [29], the minimum mean-square error (MMSE) method [5], and the multi-Doppler-direction constraint method [30]. Besides, Zuo et al. presented an improved signal reconstruction method based on Doppler spectrum estimation by Capon estimation [31]. Another process for nonuniform signal reconstruction is based on the Nonuniform Fast Fourier Transform (NUFFT), but the reconstruction efficiency of this approach is low. However, the nonuniform samples acquired by azimuth multichannel SAR have two characteristics: each channel signal is uniform and a set of samples from multiple channels is uniform. Based on the above two characteristics, the optimized NUFFT is proposed, which improves the processing performance of multichannel signal reconstruction [32].

In addition, due to the limitation of the transmission timing and the range ambiguity, when the sampling positions of different channels are spatially coincident (singular points) or close to coincident, the conventional reconstruction algorithm leads to a sharp deterioration of the system performance in terms of the SNR and azimuth ambiguity. For the case of overlapping spatial sampling locations of some channels in a multichannel system, Guo et al. proposed to divide all channels into several groups to reconstruct data separately, and finally accumulate the several reconstruction results to obtain a single-channel signal with an improved SNR [33]. When the sampling locations of different channels are close to coinciding, Liu et al. proposed the IDBF algorithm, which includes ambiguity index screening based on the equivalent sampling and effective reconstructed Doppler bandwidth, and Doppler spectrum weighting based on the maximum-to-minimum ratio of the noise spectrum [34]. Liu et al. proposed the ImpMMSE algorithm, which improves the SNR of the reconstructed system by eliminating the smaller singular points in the pre-filter bank through ambiguity index screening, and improves the azimuth ambiguity by applying the MMSE [35]. It is noted that azimuth ambiguity and noise cannot be minimized at the same time. Thus, Zhang et al. proposed a multichannel reconstruction algorithm based on a multiobjective optimization model to significantly reduce the noise of the system under the premise of guaranteeing the performance of azimuth ambiguity [36]. Cheng et al. proposed a multichannel signal reconstruction method using the Vandermonde component of the system matrix based on the least squares principle [37].

In an azimuth multichannel SAR system, by developing superior multichannel signal reconstruction methods, the azimuth ambiguity can be effectively suppressed and the SAR images can be well focused, theoretically [20,25].

### 3.3. Multichannel Error Calibration

**Figure 5:**
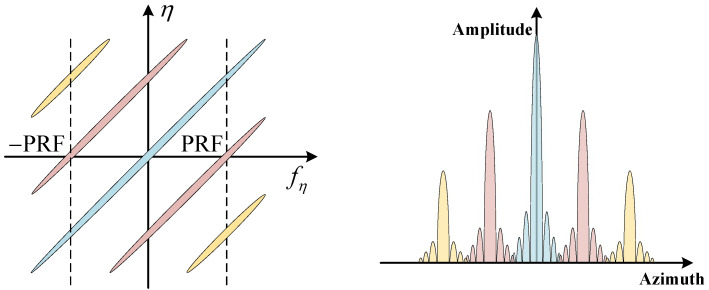
Effect caused by amplitude and phase errors in azimuth multichannel SAR system, where the left figure shows the azimuth spectrum shift schematic and the right figure shows the corresponding azimuth impulse response results, the blue color indicates the desired signal, and the other colors indicate the ambiguous signal caused by the errors.

In practical applications, due to the complexity of the azimuth multichannel configuration, multichannel errors, such as amplitude error and phase error, are inevitably generated. These errors lead to signal reconstruction filter mismatches, which further results in azimuth spectrum shift. Taking azimuth three-channel SAR as an example, Figure 5 gives the schematic of the effects caused by amplitude and phase errors. It can be seen that the shifted spectrum appears as virtual targets or azimuth ambiguity in the SAR image. Therefore, the multichannel error calibration, especially phase error calibration, is the key link for the signal reconstruction of azimuth multichannel SAR to obtain unambiguous images.

To tackle the above problem, some effective azimuth multichannel error calibration methods have been proposed. Some of these methods characterize the effect of the error by deriving an analytical expression for the phase error. Gao et al. gave analytical formulas for the position and relative amplitude of virtual targets when channel errors exist. But this method is limited to a uniform distribution of equivalent phase centers and is not universally applicable [38]. Almeida et al. proposed a residual amplitude–phase error model from a statistical point of view, and gave a quantitative formula for the effect of ambiguity on reconstructed images, which was validated by Monte Carlo experiments [39]. However, since this method is based on the statistical model, it cannot calculate the impact of an exact channel error. Xiao et al. quantitatively described the position and amplitude of virtual targets in the case of nonuniform equivalent phase center, but it is only applicable to dual-channel SAR systems, and is difficult to extend to multichannel SAR systems [40].

Moreover, the methods based on subspace are also representative. Based on the angular and Doppler ambiguity analysis of the clutter echo, the channel auto-calibration method was proposed by Li et al., where the orthogonality between the signal subspace and the noise subspace is utilized for phase estimation [41]. Further, Guo et al. proposed an improved channel error calibration method by transforming Doppler-variant covariance matrices into a constant covariance matrix to reduce the computational complexity [42]. Based on the eigen-structure method, Fan et al. proposed a robust estimation of the baseband Doppler centroid and the channel phase error [43]. Zhou et al. calculated the channel error by minimizing the MMSE of the signal subspace [44]. Sun et al. successively proposed a channel phase error calibration method based on the optimal value of the image domain quality function and an image domain subspace calibration method; the former is only applicable to dual-channel SAR, and the latter utilizes the focused data and selects the high-SNR region of the SAR image to estimate the azimuth multichannel error [45,46]. In the subspace methods above, at least one redundant channel is required for noise subspace separation, which degrades the error calibration performance when the number of Doppler ambiguities is close to the number of channels.

Two other types of channel phase error calibration methods avoid redundant channel and matrix decomposition. The first one is according to the cross-correlation. Feng et al. proposed to perform an azimuth cross-correlation operation in the range frequency domain for each channel echo, and the phase error can be derived using the linear dependence between the phase of the cross-correlation function and the range frequency [47]. A baseband Doppler centroid estimator utilizing the spatial cross-correlation coefficients of multichannel SAR was also proposed by Liu et al. [48]. The other is based on the power maximization criterion. Zhang et al. proposed a robust channel phase error calibration algorithm by maximizing the minimum variance distortionless response beamformer output power [49], and Huang et al. proposed to construct the cost function based on the power maximization criterion to obtain the channel phase errors [50]. The disadvantage of the above two types of methods is that the dependence on the Doppler center limits the estimation accuracy.

In addition, phase error estimation can be transformed into an optimization problem. Zhang et al. proposed a robust multichannel error calibration approach by the local maximum-likelihood weighted minimum entropy algorithm [51]. However, the method uses a coarsely focused image, which cannot directly obtain an ambiguity-free SAR image. Further, Xiang et al. introduced a minimum entropy channel error estimation method based on a fine-focused SAR image [52]. Liang et al. used a weighted backprojection algorithm to obtain the complex intensity of the echoes from each channel, and then estimated the channel phase errors by maximizing the image intensity using the gradient descent method [53]. A least L1-norm approach that avoids introducing computationally intensive imaging process into iterations was proposed by Cai et al. for the analysis and calibration of the channel error in the image domain [54,55]. To improve the performance of the algorithm in a strong clutter environment, Yang et al. proposed to estimate channel phase errors based on maximum normalized image sharpness [56]. To avoid the cost of establishing an objective function in the image domain leading to imaging processing in each channel, some methods have been proposed to calibrate multichannel phase error by establishing an objective function in the range–Doppler domain. A channel phase error estimation approach based on modified kurtosis maximization was proposed by Pan et al., where the objective optimization function of the reconstructed Doppler spectrum was constructed [57]. Xu et al. proposed a channel imbalance estimation method based on minimizing the sum of the sub-band norm for the reconstructed azimuth spectrum [58]. An approach termed least spectrum difference was proposed by Cai et al. to calibrate the phase error of an azimuth multichannel SAR system, which operates in the range–Doppler domain, mitigates the radio frequency interference on the objective function, and saves computational effort [59].

By adopting the above effective azimuth multichannel phase error estimation and calibration methods, the accuracy of azimuth signal reconstruction can be greatly improved, which further enhances the azimuth multichannel HRWS SAR imaging quality.

## 4. Digital Beamforming Technique

This section provides a review of the DBF technique in elevation. Section 4.1 gives the core principle of the DBF technique. Section 4.2 describes the challenge of energy loss in the DBF technique that lead to reduced gain on reception and reviews the corresponding solutions. Section 4.3 introduces the challenge faced by the DBF technique in real-time processing and the corresponding solutions.

### 4.1. Principle of DBF Technique

with the increasing demand for wide-swath imaging, a SAR system usually employs a smaller transmit antenna in elevation to achieve wide-beam coverage. However, a small transmit antenna inevitably leads to reduced transmission gain, further degrading the SNR, and the wide beam in elevation results in a sharp deterioration of the range ambiguity performance of the system. Fortunately, multichannel in elevation combined with the DBF technique can effectively solve the above problems. In this way, the antenna is divided into multiple channels in elevation, where a single aperture is used to generate a wide transmit beam to achieve wide coverage, and then each channel independently receives the echoes of the illuminated swath. All channel echo signals are time-varying weighted by the DBF technique, which is equivalent to tracking the ground echo with a dynamic high-gain narrow receiving beam, known as the scan on receive (SCORE) technique.

**Figure 6:**
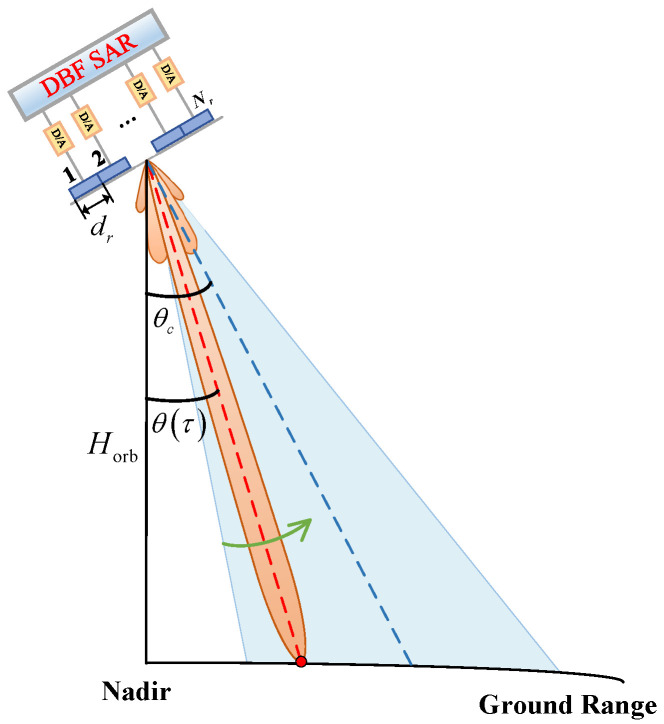
Schematic of transmit and receive signal geometry using digital beamforming (DBF) technique in elevation.

**Figure 7:**
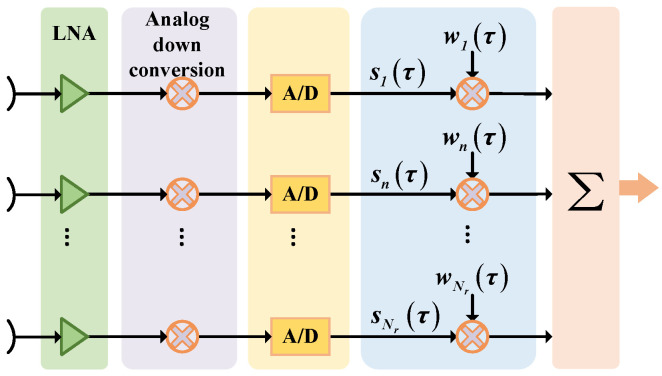
The general model for echo signal processing using the DBF technique in elevation.

Assuming that a planar antenna is used in spaceborne SAR, there are $N_{r}$ channels in elevation with a spacing of $d_{r}$, as shown in Figure 6. τ is the fast time, $\theta \left(\tau \right)$ is the look angle of the beam pointing at time τ, and $\theta_{c}$ is the tilt angle of the antenna. Taking a point target in the interested swath with a slant range of $R_{0}$ as an example, it corresponds to a look angle of $\theta_{0}$; the echo signals in range received by the *n*th channel after demodulation are given as

(19) $$s_{n}\left(\tau \right)=\mathrm{rect}\left(\frac{\tau -\tau_{n}}{\tau_{p}}\right)\cdot \exp\left\{j\pi K_{r}{\left(\tau -\tau_{n}\right)}^{2}\right\}\exp\left\{-j2\pi f_{0}\tau_{n}\right\},n=1,2,\ldots ,N_{r}$$

where $f_{0}$ is the carrier frequency and $K_{r}$ is the chirp rate. $\tau_{n}$ denotes the time delay of the echo received by the *n*th channel and can be expressed as $\tau_{n}=\tau_{0}-\Delta \tau_{n}$, $\tau_{0}$ represents the return delay of the transmitting channel, and $\Delta \tau_{n}$ is the echo time difference between the *n*th channel and the reference channel, which can be denoted as

(20) $$\Delta \tau_{n}=\frac{d_{n}\cdot \sin\left(\theta_{0}-\theta_{c}\right)}{c}$$

where $d_{n}$ is the spacing between the *n*th receive channel and the reference channel. The general model for echo signal processing using the DBF technique in elevation is shown in Figure 7, where the received beam pointing can be adjusted in real time by time-varying weighting and the weighting coefficient for the *n*th channel is

(21) $$w_{n}\left(\tau \right)=\exp\left\{\frac{-j2\pi d_{n}\sin\left(\beta \left(\tau \right)\right)}{\lambda }\right\}$$

where $\beta \left(\tau \right)=\theta \left(\tau \right)-\theta_{c}$ is the angle between the center of the received beam and the antenna. The echo signal of each channel weighted by DBF can be expressed as

(22) $$\begin{array}{c} s_{wn}\left(\tau \right)=\exp\left\{-j2\pi f_{0}\tau_{0}\right\}\cdot \mathrm{rect}\left(\frac{\tau -\tau_{n}}{\tau_{p}}\right)\cdot \exp\left\{j\pi K_{r}{\left(\tau -\tau_{n}\right)}^{2}\right\}\\ \cdot \exp\left\{j\frac{2\pi }{\lambda }d_{n}\sin\left(\beta \left(\tau_{0}\right)\right)\right\}\cdot \exp\left\{-j\frac{2\pi }{\lambda }d_{n}\sin\left(\beta \left(\tau \right)\right)\right\} \end{array}$$

The echo signals from each channel in the elevation are synthesized by the DBF technique described above, which achieves almost ideal coherent superposition and greatly improves the echo gain. The narrow beam reception can also reduce the gain of the range ambiguity signal, thus improving the RASR performance. Moreover, since the interference signal has a different direction of arrival (DOA) from the desired echo signal, the DBF technique can effectively suppress the interference signal by zeroing the received beam in a specific direction. Therefore, the DBF technique can also be used to realize echo separation.

### 4.2. SCORE Loss of DBF SAR

Although the DBF technique has great potential for improving SAR system performance, it still faces a number of challenges in its application. Among them, pulse extension loss and frequency dispersion loss result in energy loss, further deteriorating the SNR and imaging quality. To promote the development of the DBF technique, further efforts are needed to address these problems, which are reviewed in this subsection.

#### 4.2.1. Pulse Extension Loss

The narrow beam synthesized by the DBF technique has a certain width, which means the maximum gain of the echo signal only can be obtained when the target is exactly pointed by the receiving narrow beam. For a single point target, due to the modulation by the receiving beam pattern, there is a different degree of received gain loss at different echo times, which is commonly referred to as the pulse extension loss (PEL) [60,61].

**Figure 8:**
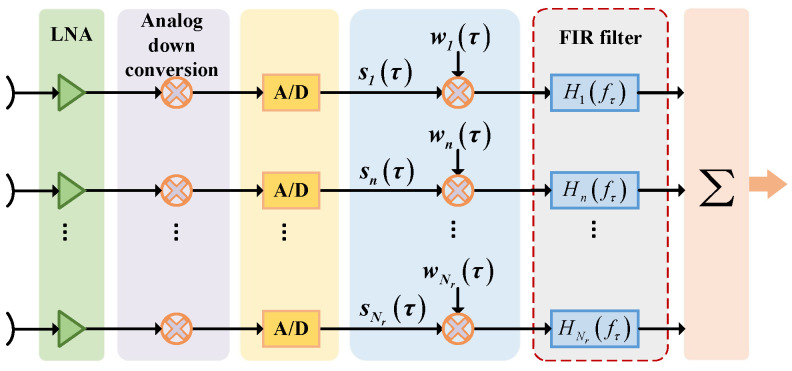
Finite impulse response (FIR) filtering method to compensate for pulse extension loss (PEL) effect.

To compensate for the effect of the PEL, Feng et al. derived the echo signal spectrum in range for each channel; thus, a linear phase that affects the coherent synthesis of the DBF can be obtained, which is dependent on the channel position and the reference slant range. Further, to compensate this phase, the finite impulse response (FIR) filtering method is proposed [62]. In this method, the data in each channel are weighted and then passed through a set of FIR time delay filters to achieve a fixed delay. The time delay in each channel is different to compensate for the effect of the PEL, and then the signals in each channel are synthesized into a signal and output. The flowchart of DBF processing for compensating the PEL effect by the FIR filtering method is shown in Figure 8. The impulse response of the FIR time delay filter for each channel can be expressed as

(23) $$H_{n}\left(f_{\tau }\right)=\exp\left\{j2\pi \frac{\left(n-1\right)\omega_{0}}{K_{r}}f_{\tau }\right\}$$

where $f_{\tau }$ is the range frequency, $\omega_{0}=\frac{d_{r}}{\lambda }\cdot \frac{\partial \beta \left(\tau \right)}{\partial \tau }{|}_{\tau =\tau_{c}}$ and $\tau_{c}$ is the echo time of the reference slant range.

In addition, Li et al. proposed several methods to mitigate the PEL such as a unified compensation processing in the frequency domain, separate compensation processing for each channel in elevation, and beamforming processing after matched filtering [63]. Among them, the weighted processing of each channel in the frequency domain is equivalent to the time delay in the time domain. However, considering that the DBF processing is required to be carried out in real time on the board, the FIR filtering method is more attractive in comparison.

The PEL problem in the DBF technique can be effectively mitigated by the above compensation methods. Thus, the energy loss caused by pulse width extension can be corrected during signal processing. This not only improves the SNR of a SAR system, but also provides more reliable technical support for HRWS SAR imaging.

#### 4.2.2. Frequency Dispersion Loss

The time-varying weighting coefficients for each channel in DBF processing are derived under the assumption of a constant frequency, i.e., the wavelength is considered to always be a constant corresponding to the carrier frequency. However, this constant wavelength approximation is only applicable with narrow-band signal models. As the demand for HRWS increases, the bandwidth of the transmitted signals of SAR systems also increases. with a large transmit bandwidth, the wavelength of the linear frequency modulated signal is no longer a constant over the entire pulse duration, which may cause the time-varying weighting coefficients processed by DBF to no longer be accurate. This results in an offset of the receiving beam pointing, which leads to a broadening of the impulse response width and a deterioration of the system SNR. This effect is known as the frequency dispersion loss (FDL).

To overcome the effect of the FDL, a Digital Scalloped Beamforming (DSBF) technique was proposed by Zhao et al. [64]. This method divides the signal into multiple narrow-band signals with different center frequencies by a set of bandpass filters, and generates corresponding sub-beams for different narrow-band signals, which is equivalent to the formation of a scalloped beam. By designing the number of filters, the beam pointing offset and energy loss problems of broadband signals can be effectively mitigated, thus eliminating the PEL and FDL. This further improves the performance of DBF synthetic signals, enhances receiving gain, and enables the DBF technique to be used for HRWS SAR imaging in complex environments.

### 4.3. Computational Load for Real-Time Processing

Theoretically, the antenna height of DBF SAR can be an arbitrary value, and DBF SAR is usually required to be equipped with multiple digital receiver channels to obtain a superior system performance (in terms of the RASR and SNR). However, many such digital receiver channels lead to a continuously increasing amount of raw data, posing new challenges with respect to on-board memory and downlink capacity. To tackle these challenges, the DBF operation has to be implemented in real time onboard to reduce the downloaded data volume, which requires a significantly huge computational load [64,65,66]. However, the computational power is a challenge for digital processors due to the extremely limited resources of spaceborne SAR, hindering the development of spaceborne DBF SAR. In addition, the implementation of compensating the PEL and FDL can also increase the computational load [67].

Motivated by mitigating the use of computational resources, some effective methods have been proposed in terms of DBF processing architecture and weight generation in recent years, which are reviewed in this subsection.

#### 4.3.1. IF-DBF Architecture

**Figure 9:**
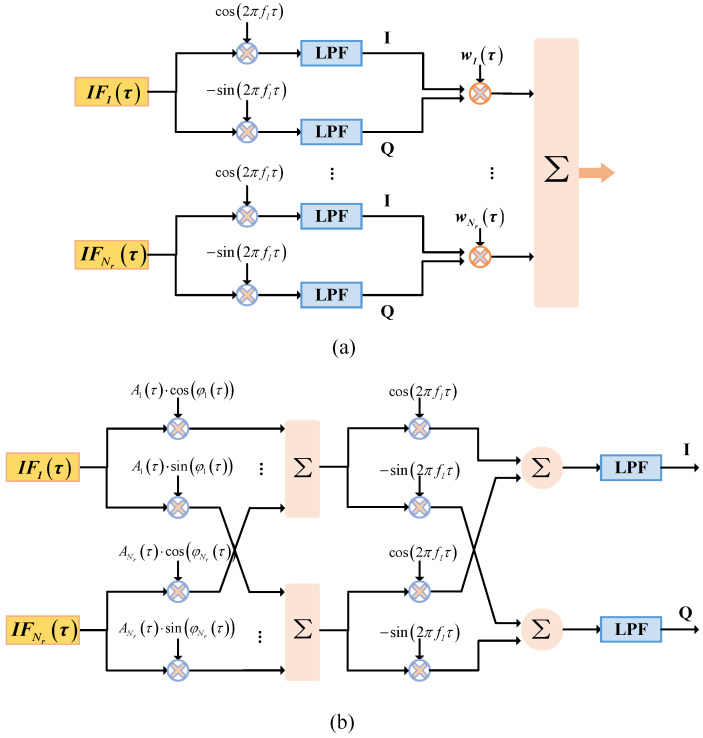
DBF digital processing architecture. (**a**) Conventional DBF processing architecture. (**b**) Intermediate-frequency (IF)-DBF processing architecture.

An advanced intermediate-frequency (IF) DBF processing architecture has been proposed by Wang et al., and a dedicated DBF processing flow suitable for the IF-DBF architecture is also provided [65]. Compared with the conventional processing architecture shown in Figure 9a, it is worth noting that DBF operations are performed at IF instead of baseband, as shown in Figure 9b. In Figure 9, $IF\left(\tau \right)$ and $f_{I}$ denote the sampled IF digital signal and IF frequency, respectively. $A\left(\tau \right)$ and $\varphi \left(\tau \right)$ represent the amplitude and phase of the complex weighting coefficient $w\left(\tau \right)$, respectively. Such an IF-DBF processing architecture has some advantages. On the one hand, due to the digital low-pass filter (LPF) most of the digital resources can be saved without degrading the DBF performance, thus significantly reducing the multiplication operation during DBF processing. On the other hand, it is compatible with some advanced processing modules, e.g., it allows introducing FIR filters related to the constant time delays to mitigate the effects of the PEL. (24) gives the number of multiplications required to perform a DBF operation in the conventional architecture.

(24) $$T_{BB}=N_{r}\left(2+2O+3\right)$$

where *O* is the order of the LPF and it is usually more than 32, the term 2 and 3 represent the number of multiplications caused by down-conversion and weighting operation. While the number of multiplications required in the IF-DBF architecture is as follows

(25) $$T_{IF}=2N_{r}+4+2O$$

It can be seen that the number of multiplications required by the conventional DBF architecture increases significantly as the number of channels increases, while the number of multiplications required by the IF-DBF architecture increases slowly. The main reason is the decoupling of the computational load due to the LPF and the number of channels in the IF-DBF architecture. To confirm the validity of the IF-DBF architecture, Meng et al. conducted experiments based on real SAR data acquired by C-band and X-band airborne DBF SAR, and demonstrated the IF-DBF SAR imaging results for the first time [68].

#### 4.3.2. Optimized Weight Generator

Moreover, weight generation is also crucial for DBF real-time processing. To obtain real-time weights computed by (21), DBF-SAR is required to perform operations such as division, inverse cosine, and sine functions in real time according to specific geometric and echo timing, which consumes huge amounts of computational resources [66]. To tackle this problem, Qiu et al. propose a parameterized and high-efficiency weight generator by using polynomial approximation, which can be described as

(26) $$w\left(\tau \right)\approx y\left(\tau \right)=\sum_{j}A_{j}\tau^{j}$$

where $y\left(\tau \right)$ is a polynomial of τ, *j* is the order of the polynomial, which is usually chosen as 4, and $A_{j}$ is the polynomial coefficient. It can be seen that the high efficiency of such a weight generation depends on two important factors. One is that the computation of weights requires only a finite number of additions and multiplications, which avoids occupying computational resources due to complex operations. Another is that the parameters $A_{j}$, which are complex but constant during the computation process, can be first provided by an auxiliary computer on the ground and then uploaded to a satellite. This effectively reduces the computational load of the real-time processors on board.

## 5. PRI Variation Technique

This section provides a review of PRI variation techniques. Section 5.1 outlines the fundamental principle of the PRI variation technique. Section 5.2 describes various PRI sequence variation strategies. Section 5.3 discusses the challenges associated with signal processing in a varied-PRI SAR system and the corresponding solutions.

### 5.1. Principle of PRI Variation Technique

**Figure 10:**
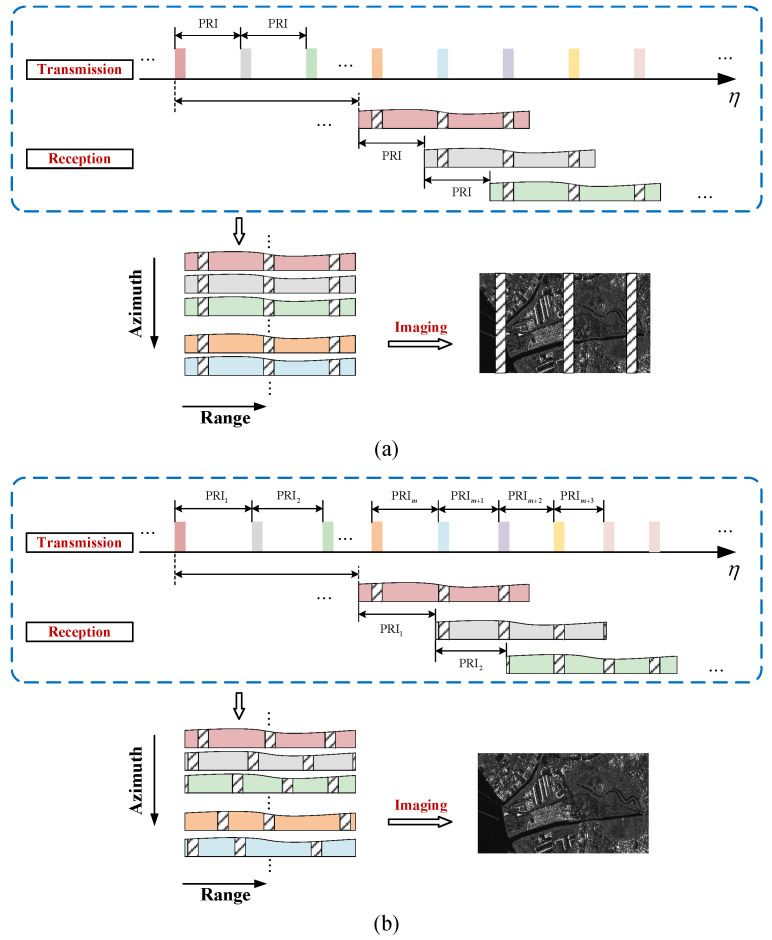
Schematic of blind range locations. (**a**) Constant PRI SAR. (**b**) Varied-PRI SAR.

As mentioned above, the combination of the elevation multibeam technique with the DBF technique enables the simultaneous reception of high-gain echoes from multiple sub-swaths, thereby achieving wide-swath SAR imaging. However, due to interruptions in reception during transmission (i.e., transmission blockage), blind ranges inevitably occur between adjacent sub-swaths. Moreover, since the PRI remains constant, the positions of the blind ranges are fixed along the azimuth. This results in continuous blind ranges in the SAR image, as shown in Figure 10a, which prevents the continuous observation of ultrawide swath [69]. To takle the above problem, the PRI variation technique has been proposed. The principle of PRI variation is to shift the corresponding blind ranges across the swath by varying the time intervals between transmitted pulses [15]. When the PRI changes continuously and periodically, the positions of the blind ranges also vary periodically. In one PRI variation cycle, the blind ranges appear at different locations in the echo windows corresponding to different transmitted pulses. This results in the loss of only a few azimuth samples at the same slant range in the interested swath, thus enabling the possibility of continuous observation over the entire swath, as illustrated in Figure 10b [69].

In particular, Villano et al. termed the SAR imaging mode with the PRI variation technique the staggered imaging mode, which is typically combined with the DBF technique to meet the requirements of ultrawide continuous swath imaging [16,70]. Staggered SAR is currently the baseline acquisition mode for Tandem-L, capable of achieving a swath width of 350 km in the single-/dual-polarimetric mode and 175 km in the quadrature-polarimetric mode, with an azimuth resolution of approximately 7.5 m. The NASA-ISRO SAR (NISAR) mission also plans to utilize the staggered mode to achieve a continuous swath of 240 km with an azimuth resolution of 6 m [18,19,71,72]. In addition, based on the PRI variation technique, the staggered ambiguous SAR mode has been proposed, which allows imaging a wide swath with a high resolution for specific ship monitoring applications without the need for the DBF technique in elevation [73].

In varied-PRI SAR, the blind ranges are still present even though they are dispersed across the swath, which inevitably leads to the loss of azimuth samples. Additionally, due to the continuous variation of PRI, the azimuth sampling is nonuniform, which prevents the raw data from being directly imaged. Consequently, the recovery of missing azimuth samples and the reconstruction of the azimuth signal are critical aspects of signal processing in varied-PRI SAR. To achieve accurate azimuth signal recovery and reconstruction, one feasible approach is to design the PRI sequence such that the position and number of blind ranges are controllable. Another approach is to develop precise signal processing methods for azimuth signal recovery and reconstruction.

### 5.2. PRI Variation Strategy

As discussed, the PRI variation strategy is one of the research focuses of the PRI variation technique. Various strategies have been proposed, including linear PRI variation, stepwise PRI variation, and pseudo-random PRI variation, among others [70,74,75]. Of these, linear PRI variation sequence offers notable advantages. It enables SAR system designers to optimize the selection of the PRI sequence in conjunction with other system parameters, such as slant range and transmission pulse duration, while providing direct control over the position and/or width of the blind range [15,70]. Additionally, advanced SAR missions, such as Tandem-L and NISAR, advocate for the use of linear PRI variation [18,19]. Accordingly, a detailed review of the linear PRI variation strategies is provided below.

**Figure 11:**
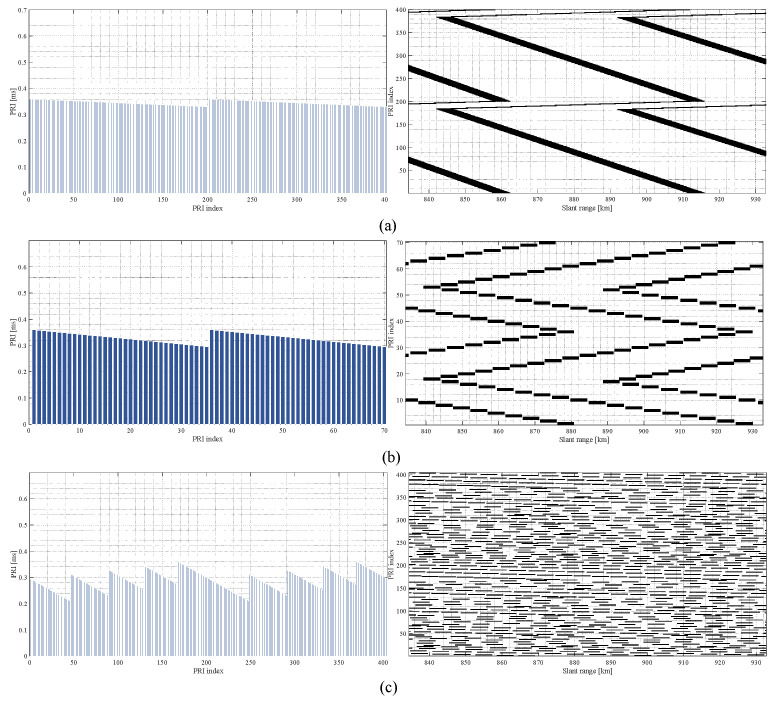
Example of linear PRI sequences, where the PRI trend is given on the left and the blind range location is given on the right. (**a**) Slow PRI change sequence. (**b**) Fast PRI change sequence. (**c**) More elaborated PRI sequence.

**Figure 12:**
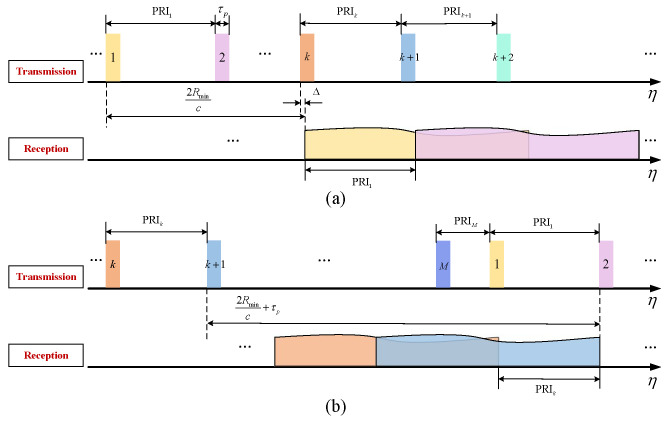
Schematic of transmitted and received pulses, the numbers indicate the indexes corresponding to the transmitted pulses in a cycle. (**a**) Graphical representation of (28) and (29). (**b**) Graphical representation of (32).

Assuming the swath of interest extends over the range $R_{\min}$ to $R_{\max}$, the corresponding echo window start and end times are $2R_{\min}/c$ and $2R_{\max}/c+\tau_{p}$, respectively. without loss of generality, a PRI sequence that linearly decreases within one cycle is considered. Assuming the sequence consists of *M* PRI values in a cycle, with a difference of Δ between adjacent PRI values, the PRI sequence can be expressed as:

(27) $${\mathrm{PRI}}_{m}={\mathrm{PRI}}_{m-1}-\Delta ={\mathrm{PRI}}_{1}-\left(m-1\right)\Delta ,m=1,2,\ldots ,M$$

where ${\mathrm{PRI}}_{1}$ represents the maximum value in the sequence. The linear PRI sequence can be further classified into slow PRI change and fast PRI change [70]. As shown in Figure 11a, a slow PRI change sequence consists of a set of longer PRI values that change more gradually. The advantage of this sequence is that it achieves a larger minimum PRI value, resulting in improved range ambiguity performance. However, due to the continuous loss of azimuth samples, slow PRI change leads to an increase in the width of the blind range in azimuth, causing high sidelobes near the main lobe after azimuth focusing [70]. As shown in Figure 11b, the fast PRI change sequence is characterized by a smaller *M* and a relatively larger Δ. The design criterion for this sequence is that any two consecutive azimuth samples are not simultaneously lost [15,70,76]. Assuming the maximum value of the PRI sequence has been determined based on the appropriate sampling interval, the method proposed by Villano et al. for calculating *M* and Δ is as follows [69]. As shown in Figure 12a, assuming the first blind range within the echo window of the first transmitted pulse is caused by the *k*th transmitted pulse, the constraint to avoid consecutive sampling losses is given by

(28) $${\mathrm{PRI}}_{1}-{\mathrm{PRI}}_{k+1}\geq \tau_{p}$$

(29) $$\sum_{m=1}^{k-1}{\mathrm{PRI}}_{m}+\Delta \leq \frac{2R_{\min}}{c}$$

Substituting (27) into (28) and (29) yields the minimum value of Δ and the maximum value of *k*, which are given by

(30) $$\Delta \geq \Delta_{\min}=\frac{\tau_{p}}{k_{\max}}$$

(31) $$k\leq k_{\max}=\left\lfloor \frac{\frac{2R_{\min}}{c}+{\mathrm{PRI}}_{1}-\frac{3\tau_{p}}{2}}{{\mathrm{PRI}}_{1}-\frac{\tau_{p}}{2}}\right\rfloor$$

where $\left\lfloor \cdot \right\rfloor$ is the floor function. Then, it is assumed that the last blind range within the echo window of the *l*th transmitted pulse is caused by the first transmitted pulse of the next cycle, as shown in Figure 12b. Given the linear decrement characteristic of the PRIs and (28), it is necessary to maintain l≥k+1. To achieve better range ambiguity performance, a large ${\mathrm{PRI}}_{M}$ is desired. Therefore, *l* is set to k+1 for a smaller *M*. This constraint can be expressed as follows:

(32) $$\frac{2R_{\max}}{c}+\tau_{p}\leq \sum_{m=k+1}^{M}{\mathrm{PRI}}_{m}+{\mathrm{PRI}}_{1}$$

Thus, the minimum value of *M* that satisfies (32) can be determined by Equation (33).

(33) $$M\geq M_{\min}=\left\lceil \frac{\left({\mathrm{PRI}}_{1}+\frac{\Delta }{2}\right)-\sqrt{{\left({\mathrm{PRI}}_{1}+\frac{\Delta }{2}\right)}^{2}-2\Delta \left(\frac{2R_{\max}}{c}+\tau_{p}+\left(k-1\right)\left({\mathrm{PRI}}_{1}-\frac{\Delta }{2}k\right)\right)}}{\Delta }\right\rceil$$

where $\left\lceil \cdot \right\rceil$ is the ceiling function. In particular, the German satellite TerraSAR-X can offer 512 different available PRIs and allows the transmission of pulses using *M* distinct PRIs in a periodic manner. Based on the characteristics of fast PRI change, a set of fast PRI change sequences can be selected for TerraSAR-X, ensuring that two consecutive azimuth samples are never lost. Ultimately, TerraSAR-X successfully acquired data over Lake Constance in staggered mode [69]. Similarly, the TerraSAR-X satellite conducted experimental acquisitions in staggered ambiguous mode at the North Sea test site near the German Bight, achieving staggered ambiguous mode imaging with a 2.2 m azimuth resolution over a 110 km ground swath [73].

The fast PRI change sequence avoids the issue present in slow PRI change, i.e., the elimination of high sidelobes near the main lobe in the azimuth impulse response. However, due to periodic data loss and signal processing errors, distant sidelobes may still appear in the azimuth impulse response of the fast PRI change sequence. To tackle this problem, Villano et al. further proposed a more elaborated sequence consisting of multiple different fast PRI change sequences, as illustrated in Figure 11c. This introduces some irregularities into the PRI sequence, allowing sidelobe energy to spread along the azimuth [70]. The German Aerospace Center (DLR) obtained equivalent staggered SAR data using highly oversampled F-SAR airborne data, which can meet the design requirements of various PRI sequences. Experimental results demonstrated that, under the same sampling rate and signal processing methods, more elaborated sequences achieve smaller sampling estimation errors compared to fast PRI change [69]. Therefore, as an extension of fast PRI change, the more elaborated sequences are preferred in PRI variation SAR systems, such as those planned for Tandem-L and NISAR, which propose employing more elaborated sequences for the staggered imaging mode [18,19].

Furthermore, when a SAR system uses fast PRI change with a low azimuth sampling rate, a situation occurs where only one known sample is available between two missing samples, i.e., local undersampling. This can lead to localized under-information and is not conducive to recovering missing data. Thus, Zhou et al. proposed a stepwise PRI variation strategy for low-oversampled SAR, in which around half of the raw echo data are sampled uniformly along the azimuth. with this PRI sequence, at most one missing sample occurs in each PRI variation cycle at any slant range in the interested swath, and the missing samples appear periodically along the azimuth with the PRI variation cycle. This strategy disperses the blind ranges more uniformly and can reduce errors during resampling in azimuth [74].

In addition, random sampling is another commonly used sampling strategy that can be used in SAR to achieve random PRI variation. In practice, such PRI sequences are usually pseudo-random sequences constrained by the performance of the SAR system. SAR designers design random PRI sequences using a series of constraints deliberately. Yang et al. proposed the use of Poisson disk sampling in azimuth to ensure that the interval between any two neighboring pulses is greater than the Nyquist sampling interval, thus obtaining wider-swath SAR imaging [77]. The nonuniformly undersampled raw data can be obtained by this method, and sparse reconstruction theory needs to be utilized to recover the original signal from Poisson-disk-sampled data. Similarly, Dong et al. also proposed the use of randomly varied PRIs to achieve HRWS SAR imaging. The selection of random PRIs is affected by the transmission blockage and nadir echo interference in spaceborne SAR. And this method does not require continuous changes in the interval between adjacent transmitted pulses, reducing the difficulty of hardware design for the onboard SAR sampler [75]. The essence of this method also is to improve the swath width by decreasing the azimuth sampling rate, requiring sparse reconstruction of the raw data to ensure a better azimuth resolution. Although this approach can achieve HRWS imaging through a single channel, this also limits its ability to achieve ultrawide swath SAR imaging. Moreover, Wu et al. proposed to utilize the exceptional information extraction capability of deep neural networks to separate the effective information, so that more information of interest can be obtained using as few pulses as possible. Such nonuniformly undersampled PRI sequences can be obtained by jointly optimizing the sampling pattern and imaging process to ensure imaging quality [78].

In summary, the purpose of the PRI variation strategy is to obtain the desired blind range distribution, which further enhances the SAR imaging performance or system performance. This means that arbitrary design of PRI variation rules is allowed under aspecific criterion.

### 5.3. Signal Processing

For a SAR system using the PRI variation technique, the received signal of a point target at $R_{0}$ after demodulation can be expressed as

(34) $$\begin{array}{c} s\left(\tau ,\eta \right)=A_{0}\cdot W_{r}\left(\tau -\frac{2R(\eta )}{c}\right)\cdot W_{a}\left(\eta -\eta_{c}\right)\cdot W_{b}\left(\tau ,\eta \right)\\ \cdot \exp\left\{j\pi K_{r}{\left(\tau -\frac{2R(\eta )}{c}\right)}^{2}\right\}\cdot \exp\left\{-j4\pi \frac{R(\eta )}{\lambda }\right\} \end{array}$$

where $A_{0}$ is a complex constant representing the signal backscattering coefficient, η is the nonuniform azimuth sampling time, and $\eta_{c}$ is the offset time of the beam center in azimuth. $R\left(\eta \right)=\sqrt{{R_{0}}^{2}+{\left(v_{s}\eta \right)}^{2}}$ is the instantaneous slant range. $W_{r}\left(\tau \right)$ and $W_{a}\left(\eta \right)$ are the elevation beam pattern and the azimuth beam pattern, respectively. $W_{b}\left(\tau ,\eta \right)$ is a rectangular window as a function of time, describing missing data caused by transmission blockage. Obviously, the PRI variation technique inevitably causes the losing of samples and nonuniform sampling in azimuth. Thus, varied-PRI SAR faces two challenges in signal processing, i.e., recovering the missing sample data and reconstructing the nonuniform samples of the raw echoes, which directly affects the imaging quality and the ambiguity performance of the SAR system. Therefore, this subsection reviews some successive proposed methods for signal processing in varied-PRI SAR.

Villano et al. proposed the use of the azimuth multichannel reconstruction algorithm mentioned in Section 3.2 for varied-PRI SAR data reconstruction. In this method, the available samples at each range are equated to an alternating arrangement of samples from multiple channels, and each channel is uniformly undersampled. Then, the equivalent uniformly sampled signal can be obtained by reconstruction filters [16]. In this way, the number of lost samples at each range is different, resulting in a different number of samples at each range after reconstruction. As a result, the reconstructed signal needs to be processed by segmented imaging [76]. Luo et al. proposed improved multichannel reconstruction algorithms for PRI variation SAR data reconstruction, which minimize the residual ambiguity and noise power by the linear constrained minimum power (LCMP) method to improve the quality of reconstruction [79]. However, since signals outside the Doppler bandwidth are aliased into the bandwidth, multichannel reconstruction of variable-PRI SAR data suffers from reconstruction errors, and the reconstruction errors increase, especially when the number of channels is large, i.e., the PRI sequence is too long.

Another option is Best Linear Unbiased (BLU) estimation proposed by Villano et al. BLU exploits the statistical properties of SAR echo signals for the uniform reconstruction of azimuth samples, effectively improving the interpolation accuracy [70]. The core principle of BLU estimation is to use the Power Spectral Density (PSD) information corresponding to the azimuth antenna pattern to estimate the unknown samples by known samples. Assuming that the azimuth raw signal of SAR with variable PRI is $s_{0}\left(\eta \right)$, $S_{0}\left(f\right)$ is the spectrum of $s_{0}\left(\eta \right)$, the PSD $P_{s}\left(f\right)$ can be expressed approximately as

(35) $$P_{s}\left(f\right)={\left|S_{0}\left(f\right)\right|}^{2}$$

**Figure 13:**
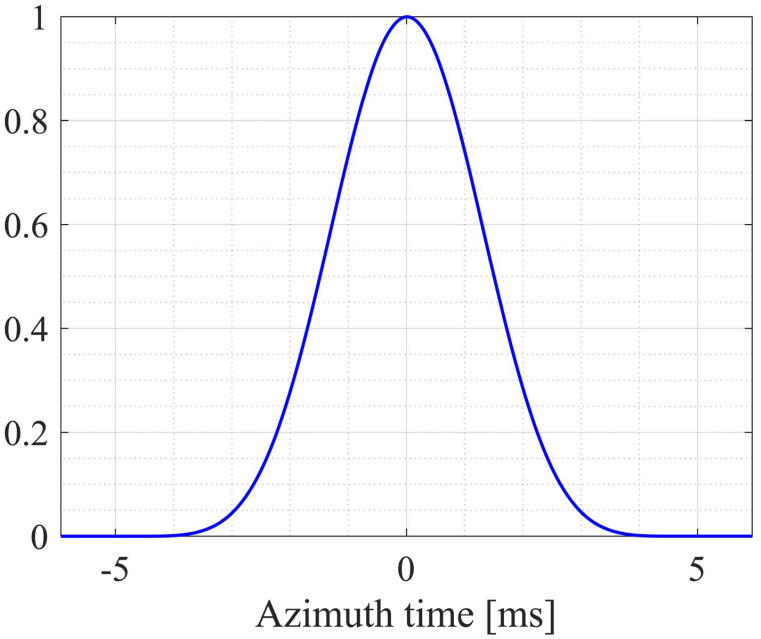
Example of the normalized autocorrelation function curve for a typical spaceborne SAR azimuth signal.

According to the Wiener–Khinchin theorem, the autocorrelation function of $s_{0}\left(\eta \right)$ can be expressed as

(36) $$R_{s}\left(\eta \right)=\mathrm{IFFT}\left[P_{s}\left(f\right)\right]$$

when an uniformly illuminated antenna in azimuth is used; the normalized autocorrelation function curve for a typical spaceborne SAR azimuth signal is given in Figure 13. It can be seen that the $R_{s}\left(\eta \right)$ is not zero only when the time interval between the SAR azimuth samples is less than a certain threshold. Assuming that the sample $s_{0}\left(\eta_{0}\right)$ at time $\eta_{0}$ is estimated, u is a column vector consisting of correlated samples $s_{0}\left(\eta_{q}\right)\left(q=1,\ldots Q\right)$, i.e., *Q* known samples are used to estimate the sample $s(\eta_{0})$. r and g denote the cross-covariance column vector and covariance matrix, respectively; the best linear unbiased estimation for $s(\eta_{0})$ can be denoted as

(37) $$s_{0}\left(\eta_{0}\right)=u^{T}g^{-1}r$$

It is easy to find that BLU can accurately estimate the azimuth samples when the oversampling in azimuth is high enough. Additionally, for the equivalent staggered SAR data obtained from highly oversampled F-SAR airborne data, DLR researchers further applied BLU estimation to resample the data onto a uniform grid. Experimental results demonstrated that as the sampling rate increased, the estimation accuracy of BLU estimation could be improved [69]. However, when SAR has low oversampling, the insufficient number of correlated samples leads to a degradation of estimation accuracy, and strong targets produce image artefacts.

For accurate recovery of the azimuth samples with low oversampling in PRI variation SAR, Wang et al. proposed the missing-data iterative adaptive approach (MIAA). This method estimates the complete spectrum by adaptive iteration and then recovers the missing data by the weighted least squares method [80]. To verify the effectiveness of the MIAA, Wang et al. generated equivalent varied-PRI SAR data from observations acquired by the HJ-1-C spaceborne SAR satellite over the city of Wenzhou, China. The results validated that under low oversampling rates, the MIAA can achieve fine imaging quality [80]. However, the quality of missing data recovery for extended targets is degraded due to the fact that the MIAA relies on the degree of predictability of the signal. In contrast, BLU can achieve optimal linear estimation of the missing data of uniform distributed targets by the least squares method. Therefore, combining BLU and the MIAA can further improve the data recovery capability [18,81]. In addition, for low-oversampled PRI variation SAR data recovery, Zhou et al. proposed several linear Bayesian methods for estimating missing data in PRI variation SAR, including Linear Bayesian Prediction (LBP), Corrected LBP (CLBP), Linear Bayesian Estimation (Linear Bayesian Estimation, LBE), and Improved LBE (ILBE). Simulations demonstrate that the flexible prior information based on the scene in linear Bayesian estimation methods can be adapted to a variety of scatterers, leading to high-quality data recovery. LBE performance outperforms BLU regardless of the oversampling, but the linear Bayesian approach is only applicable to range-compressed data, since the prior information can only be accurately estimated by increasing the SNR [74]. Liu et al. proposed a compressive sensing image reconstruction method for low-oversampled SAR, in which a hybrid-domain model of low-oversampling SAR echoes is given, and then the image reconstruction is realized by the 2D fast iterative shrinkage thresholding algorithm (ISTA) [82]. Further, Chen et al. proposed a time-domain data reconstruction algorithm based on Bayesian compressed sensing (BCS) for low-oversampled SAR. BCS applies a hierarchical form of conjugate prior to reconstruct imaging results and suppress high sibelobes in azimuth [83]. Huang et al. proposed to use a modified ε-insensitive loss tube regression and L2 regularization method to recover missing data, and then use a generalized scale transform to reconstruct the azimuth samples uniformly. Furthermore, Huang et al. utilized airborne SAR data from a region in Xi’an, China, and the Chinese spaceborne Genfen-3 SAR system to simulate varied-PRI data respectively, thereby validating the effectiveness of the proposed method. Experimental results demonstrated that this method can effectively recover the missing data with relatively low oversampling and improve the data recovery accuracy in the case of a low SNR [84]. Moreover, the NUFFT interpolation method has been proposed to resample the nonuniform data in PRI variation SAR [85]. However, simulations show that the signal reconstruction performance using NUFFT directly is usually unsatisfactory. Therefore, Wu et al. proposed to incorporate a NUFFT matched-filter operator into a deep learning network-based SAR imaging framework for sparse SAR imaging [78]. Liu et al. proposed a sparse staggered SAR imaging method based on compound regularization and NUFFT. The method permits the suppression of azimuth ambiguity without data recovery by creating a data loss index matrix [86].

To reduce the computational complexity of varied-PRI SAR signal processing, Xu et al. proposed a method that achieves uniform azimuth sampling by adjusting the phase center. In this mode, the effective phase centers of both the transmitting and receiving antennas are periodically adjusted, allowing for direct access of uniformly distributed azimuth samples. However, this method imposes strict constraints on the PRI variation strategy [87].

By utilizing the above preprocessing methods, the raw azimuth signal can be recovered and reconstructed. Then, the HRWS continuous SAR image can be obtained by using conventional SAR imaging algorithms directly [88].

## 6. Discussion

In this paper, a succinct review of the azimuth multichannel technique, the DBF technique, and the PRF variation technique are provided separately. All three techniques play important and different roles in achieving HRWS imaging. To understand the three techniques in more depth, the comparisons among the three techniques are given in this section in terms of the advantages and disadvantages .

(1) Azimuth multichannel technique

Advantages: It provides additional azimuth phase centers by spatially sampling, thus acquiring an equivalent PRF that is several times the system PRF. Consequently, the inherent contradiction between swath width and azimuth resolution is significantly mitigated. Another advantage is that the azimuth multichannel technique has been developed into a reliable engineering technique that allows the quality factor (i.e., the ratio of swath width and azimuth resolution) of on-orbit spaceborne SAR systems to exceed 10.

Disadvantages: Azimuth signal reconstruction is generally required to obtain uniform samples. On the one hand, to obtain good reconstruction performance, it is required that the amplitude and phase are consistent between the channels, and the choice of the PRF is also limited by satellite speed and antenna size. On the other hand, due to the errors in azimuth signal reconstruction, it has the potential risk of worsening azimuth ambiguity performance and the SNR. In addition, with the azimuth multichannel technique alone it is difficult to achieve ultrawide swath (200 km) due to high costs and the requirement for ultralong antenna (20 m) [89].

(2) DBF technique

Advantages: It can form a dynamic pencil beam to follow the ground echo and thus obtain a high receiving gain. By using the DBF technique, the height of the receiving antenna is independent of the swath width, allowing high system sensitivity and excellent RASR performance over the entire swath. In addition, the DBF technique provides higher angular freedom in elevation; thus, it can be utilized for echo separation, RF interference suppression, etc.

Disadvantages: The amplitude and phase of each channel are required to be the identical, otherwise the DBF synthesis of multichannel signals becomes ineffective. In addition, it inevitably leads to significantly increased hardware complexity of the system and a huge computation load due to real-time DBF processing, which hinders the further development of the spaceborne DBF technique.

(3) PRF variation technique

Advantages: By continuous variation of the PRI, the blind ranges caused by transmission blockages can be shifted across the interested swath so that most of the samples at each slant range can be obtained. The PRI variation technique breaks the constraint of the PRI on the echo window length, which in turn provides a high potential for achieving ultrawide continuous swath SAR imaging with an appropriate antenna length.

Disadvantages: It has to solve the two inherent unavoidable problems: the loss of the echo signal and the nonuniform sampling in azimuth. Moreover, to limit the additional ambiguous energy caused by signal processing, the mean PRF on transmit of the PRI variation SAR system has to be higher than the PRF of constant PRI SAR, which may lead to an increase in the amount of data.

Further, the above three techniques can be combined to achieve a higher imaging performance of the SAR system, e.g., the HRWS SAR imaging mode combines the azimuth multichannel technique and the DBF technique in elevation, and Tandem-L proposes to use the DBF technique and the PRI variation technique as the baseline acquisition mode, which is also an attractive solution for the NISAR mission [10,18,19]. Further, Almeida et al. proposed to combine the staggered mode with the azimuth multichannel technique to improve the azimuth resolution of a SAR system [90].

## 7. Perspectives

HRWS SAR has played an significant role in remote sensing for decades, providing high-performance, day-and-night, and weather-independent imaging capabilities. It has a wide range of applications, including environmental and Earth system monitoring, planetary exploration, terrain mapping, deformation monitoring, geoscience, and climate change research. with the growing demand for high-resolution and timely geospatial information, HRWS SAR continues to be a critical tool due to its distinctive advantages, whether used independently or as part of a space-based sensor network. Based on the observation requirements of SAR users, this section provides a brief perspective on the future development of HRWS SAR.

(1) Ultra-High Resolution and Ultra-Wide Swath: Current HRWS SAR enables either ultrawide swath imaging (with swath widths of several hundred kilometers and meter-level resolution) or ultra-high resolution (with swath widths of tens of kilometers and sub-meter resolution). For instance, the recently launched Advanced Land Observing Satellite-4 (ALOS-4) provides a resolution of 3 m with a swath width of 200 km [91], while the Capella SAR achieves a resolution of 0.3 m with a swath width of 5 km [92]. Given the significantly increasing demand for detailed and timely target information in remote sensing applications, it is reasonable to anticipate that future HRWS SAR systems will aim to achieve both ultra-high resolution and ultrawide swath capabilities simultaneously. Ultra-high resolution and ultrawide swath (UHR-UWS) SAR will outperform the imaging capacity of current SAR by at least one order of magnitude and allow the global observation of dynamic processes on the Earth’s surface with hitherto unknown quality and resolution.
(2) Distributed HRWS SAR: The integration of distributed SAR with HRWS SAR is an increasingly prominent area of research. As the demand for HRWS grows, monostatic HRWS SAR faces significant challenges, including increased system complexity, weight, volume, and limited platform resources. Distributed SAR offers an attractive solution to these challenges. By deploying multiple transmitters and/or receivers on different platforms, distributed SAR can capture multi-angle scattering information and provide flexible baseline configurations. These capabilities enhance digital elevation model (DEM) inversion, moving target detection, and velocity estimation. Current on-orbit distributed SAR systems, such as HT-1 [93], have demonstrated these advantages, and upcoming missions like Harmony and Tandem-L [72,94] are expected to achieve similar benefits. The future of distributed HRWS SAR lies in its ability to accomplish a variety of observation tasks through the application of advanced formation flying techniques, synchronization methods, and multi-static signal processing techniques [95,96].
(3) Multiple Imaging Modes: To accommodate various observation tasks and enhance the operational flexibility of SAR, HRWS SAR is required to support multiple imaging modes simultaneously. These modes include stripmap, scan, sliding, spotlight, and varied-PRI modes, among others, which can be switched seamlessly. Current HRWS SAR, such as GF-3 and LuTan-1, already possess the capability to operate in multiple imaging modes [6,8]. Furthermore, emerging technologies like Concurrent SAR [97,98] enable simultaneous imaging of multiple regions in different modes. In addition, HRWS SAR will still be required to have the ability to acquire multiple polarizations and multiple frequency bands to extract higher dimensional target information. Looking forward, HRWS SAR is expected to continue this trend and extract more comprehensive information through the joint processing of SAR data in various modes.
(4) Combination of advanced techniques: There is no doubt that the combination of SAR with various advanced techniques is pivotal to the development of HRWS SAR. For example, HRWS SAR can leverage some novel signal processing techniques to enhance system performance, such as random sampling [75], coprime sampling [99], deep learning techniques [78], etc. Additionally, HRWS SAR benefits from advanced antenna techniques, including frequency scanning antennas [100,101,102], frequency diversity array antennas [103], and large reflector antennas combined with digital beamforming technology [104]. These innovative theories and techniques are essential for fully exploiting the observational potential of HRWS SAR and enhancing its effectiveness in remote sensing applications.

## 8. Conclusions

This paper provides a review on the latest development of HRWS SAR systems in term of the three classical and important techniques, i.e., the azimuth multichannel technique, DBF technique, and PRI variation technique. Each technique is reported with a focus on the basic concept, operating principle, challenges, and corresponding solutions. Looking back to the 1950s, the SAR imaging technique was proposed for the first time and then there was a big expectation about the future of radar remote sensing. In the 1990s, an important shift occurred in SAR development from the technology drive due to the user demand pull. From the 2010s, spaceborne HRWS SAR has entered into a golden age of development. Looking now at the state of the art of SAR systems and applications, there is no doubt that HRWS SAR has been and will remain an integral part of Earth remote sensing since it is the only technology that can provide all-weather, day-and-night, high-resolution, and timely geospatial information with global access and coverage.

Future HRWS SAR concepts might remains as the three major technique elements or follow the similar core ideals. In addition, the three techniques can be combined with some novel signal processing theories, advanced antenna techniques, constellations, etc., to improve the imaging capability of current HRWS SAR systems by at least an order of magnitude. These highly innovative concepts will allow spaceborne SAR to achieve wide and frequent dynamic observations of the Earth at a fairly high resolution.

## Data Availability

No new data were created or analyzed in this study. Data sharing is not applicable to this article.
